# Predictive factors for readmission after bariatric surgery: Experience of an obesity center

**DOI:** 10.1097/MD.0000000000039242

**Published:** 2024-08-09

**Authors:** Mohammad Rashdan, Lana Al-Sabe, Mohammad Salameh, Shahed Halaseh, Bana Al-Mikhi, Shereen Sha’bin, Lina Alqirem, Tabarak Alsaadi, Jood Ahmad, Aseel Sabbagh, Faris Haddad, Yasser Algharrawi, Laith Alghazawi, Mohammad N. Nofal

**Affiliations:** a Department of General Surgery, Faculty of Medicine, The University of Jordan, Amman, Jordan; b Faculty of Medicine, The University of Jordan, Amman, Jordan; c Department of Surgery and Cancer, Imperial College London, London, United Kingdom.; d Department of General Surgery, Faculty of Medicine, Mutah University, Al Karak, Jordan

**Keywords:** hospital readmission, Laparoscopic Roux-en-Y Gastric Bypass, laparoscopic sleeve gastrectomy, predictors of readmission

## Abstract

Avoidable readmissions after bariatric surgery are a major burden on the healthcare systems. Rates of readmission after bariatric surgery have ranged from 1% up to 20%, but the factors that predict readmission have not been well studied. The objective of this study was to determine readmission rates following bariatric surgery and identify factors that contribute to early (within 90 days of surgery) and late readmission. A retrospective cohort study of 736 patients undergoing either Laparoscopic Sleeve Gastrectomy or Laparoscopic Roux-en-Y Gastric Bypass in Jordan University Hospital from 2016 to 2019. Demographic characteristics, co-morbidities, and readmissions were extracted from their medical records and analyzed. Multivariable logistic regression analysis was performed to determine which factors predict readmission. A total of 736 patients had bariatric surgery (Laparoscopic Sleeve Gastrectomy 89% vs Laparoscopic Roux-en-Y Gastric Bypass 11%) during the study period. Thirty-day readmission rate was 6.62% and an overall readmission rate of 23.23%. Common reasons for early readmission (within 90 days of surgery) were nausea, vomiting, and dehydration. Late readmissions were mainly caused by gallbladder stones. Three risk factors were identified that independently predicted readmission: the type of procedure being performed (*P*-value = .003, odds ratio [OR] 2.14, 95% confidence interval [CI] 1.32–3.49), depression (*P*-value = .028, OR 6.49, 95% CI 1.18–52.9) and preoperative body mass index (*P*-value = .011, OR 1.03, 95% CI 1.01–1.05). Several factors were identified that cause patients to represent and subsequently admitted into hospitals. Early readmission was usually due to nausea, vomiting, and dehydration, whereas late admissions were mostly due to biliary complications. Preoperative body mass index and depression were independent risk factors for readmission.

## 1. Introduction

Obesity epidemic has become a major public health concern globally,^[1]^ increasing the popularity of surgical management over dietary solutions. Patients who have a body mass index (BMI) greater or equal to 35 kg/m², regardless of presence, absence, or severity of co-morbidities are considered potential candidates for bariatric surgery.^[2]^ Bariatric surgery remains the only effective long-term weight loss modality for obese individuals with morbid obesity and it is associated with substantial improvement in obesity-related comorbidities.^[3]^ As a result, bariatric surgery is growing exponentially around the world.^[4]^

Debate remains regarding the most effective surgical procedure but both Laparoscopic Sleeve Gastrectomy (LSG) and Laparoscopic Roux-en-Y Gastric Bypass (RYGB) and are well established surgical modalities with low morbidity and mortality rates.^[5–7]^ However, early readmission after bariatric surgery remains a prevalent problem with rates varying between 1% to 20%, depending on the cohort size and procedure type.^[8]^ Several studies demonstrate that RYGB is associated with the greatest readmission rate, followed by LSG.^[9,10]^

Readmission following bariatric surgery remains a burden on healthcare and nearly 50% of early readmissions are potentially preventable.^[11]^ Therefore, understanding the underlying reasons for readmission is essential and may aid healthcare providers to target their efforts to reduce avoidable early readmission rates.^[12]^ In this study, we analyzed predictors of readmission in a single center in Jordan from 2016 to 2019. Our primary aim was to identify independent predictors and risk factors of readmission. Secondary aims were to determine hospital readmission rates and their underlying causes.

## 2. Methods

### 2.1. Study design

A retrospective cohort study was conducted on patients who underwent bariatric surgery between 2016 and 2019 at Jordan University Hospital (a single center). Procedures include both LSG and RYGB. Specific inclusion and exclusion criteria were set prior to data collection, described below. Prospectively collected data were extracted from the hospital’s medical records. Multivariable logistic regression analysis was performed to determine which factors predict readmission.

### 2.2. Inclusion criteria

1. The procedure performed was either LSG or RYGB.
2. All patients who underwent surgery between January 2016 and December 2019.
3. Adult patients; identified as 18-years of age and above.

### 2.3. Exclusion criteria

1. Patients <18-years old.
2. Previous history of bariatric surgery.
3. Pregnancy.
4. Patients with active malignancy.

### 2.4. Surgical techniques

Routine closure of the mesenteric defects during RYGB using a stapler device (Endohernia stapler).

### 2.5. Information collected

Data gathered included were specifically: patient’s age, gender, patient comorbidities (including and not limited to hypertension, ischemic heart disease, deep vein thrombosis, obstructive sleep apnea, pulmonary embolism, asthma, peptic ulcer disease, depression, and arthritis, etc), BMI status (preoperative and postoperative), postoperative weight (postoperatively after 6 months and lowest recorded weight), eating habits (sweet, night, emotional, and binge eating), smoking status (current smoker, ex-smoker, secondhand smoker, and nonsmoker), operation type (LSG vs RYGB), postoperative details (drain insertion, blood transfusion, and oral intake tolerance).

### 2.6. Statistical analysis

All the analyses have been conducted on R software with the proper package for each test. Categorical variables have been evaluated using frequency and X2 test to compare between different groups whereas continuous variables have been evaluated using mean and standard deviation. In addition, the Shapiro-Wilks test has been used to decide if the variable is normally distributed or not, for non-normally distributed variables a logarithmic convergence has been implanted and adjusted to convert it to be normally distributed. An Independent one-sample T-test has been used to compare continuous variables between 2 groups. Multi-logistic regression analysis was used to determine factors predictable for readmission. A *P*-value <.05 was considered statistically significant for all tests.

## 3. Results

### 3.1. Baseline characteristics of the sample population

A total of 736 patients who underwent bariatric surgery were identified. Of those, 561 were females and 175 were males. The mean age of the included patients at the time of surgery was 36.3 ± 10.6 years. The mean preoperative BMI was 45.1 ± 8.28 kg/m^2^ and the lowest weight after surgery was 77.8 ± 15.7 kg/m^2^. Surgical approach was LSG in the majority of patients (n = 655; 89.0%), whereas RYGB was performed only for 81 patients (11.0%). The mean weight in 6 months postoperatively was 89.7 ± 19.1 kg/m^2^. These characteristics are summarized in Table 1.

**Table 1:** Patient characteristics, BMI and operative details.

Characteristics	Overall	No readmission	Readmission	*P*-value
	N = 736	N = 565	N = 171	
*Gender and age*				.037
Female	561 (76.2%)	420 (74.3%)	141 (82.5%)	
Male	175 (23.8%)	145 (25.7%)	30 (17.5%)	
Age at time of surgery	36.3 (10.6%)	36.8 (10.6%)	34.7 (10.4%)	.026*
*BMI and weight*				
Preoperative BMI	45.1 (8.28%)	44.6 (8.43%)	46.5 (7.68%)	.011*
Postoperative BMI	31.1 (6.57%)	31.0 (5.89%)	31.3 (8.13%)	.795
Weight in 6 months	89.7 (19.1%)	89.7 (18.5%)	89.7 (20.8%)	.988
Lowest postoperative weight	77.8 (15.7%)	78.0 (15.9%)	77.2 (15.3%)	.696
*Type of surgery*				.003*
RYGB	81 (11.0%)	51 (9.03%)	30 (17.5%)	
LSG	655 (89.0%)	514 (91.0%)	141 (82.5%)	
*Postoperatively*				
Drain inserted	8 (1.17%)	4 (0.76%)	4 (2.48%)	.093
Blood transfusion required	3 (0.44%)	2 (0.38%)	1 (0.62%)	.554
Oral intake tolerated	667 (97.7%)	508 (97.3%)	159 (98.8%)	.383

BMI = body mass index, LSG = laparoscopic sleeve gastrectomy, RYGB = Laparoscopic Roux-en-Y Gastric Bypass.

* Chi-square test is significant at α = 0.05.

Amongst all comorbidities demonstrated illustrated in Table 2, hypertension was the highest rate at 19.0% (n = 140), closely followed by obstructive sleep apnea 18.9% (n = 139). Diabetes was demonstrated in 11.0 % (n = 81) of the cohort.

**Table 2:** Patient comorbidities as well as eating and smoking behaviours.

	Overall	No readmission	readmission	*P*-value
	N = 736	N = 565	N = 171	
*Comorbidities*				
Hypertension	140 (19.0%)	105 (18.6%)	35 (20.5%)	.661
Ischemic heart disease	8 (1.09%)	6 (1.06%)	2 (1.17%)	1.000
Deep vein thrombosis	6 (0.82%)	3 (0.53%)	3 (1.75%)	.142
Obstructive sleep apnea	139 (18.9%)	109 (19.3%)	30 (17.5%)	.689
Asthma	33 (4.48%)	26 (4.60%)	7 (4.09%)	.944
Thyroid disease	58 (7.88%)	40 (7.08%)	18 (10.5%)	.192
Diabetes	81 (11.0%)	56 (9.91%)	25 (14.6%)	.113
Peptic ulcer disease	4 (0.54%)	2 (0.35%)	2 (1.17%)	.232
GERD	81 (11.0%)	60 (10.6%)	21 (12.3%)	.639
PCOS	40 (5.43%)	28 (4.96%)	12 (7.02%)	.396
Pulmonary embolism	14 (1.90%)	10 (1.77%)	4 (2.34%)	.749
Arthritis	22 (2.99%)	19 (3.36%)	3 (1.75%)	.409
Depression	6 (0.82%)	2 (0.35%)	4 (2.34%)	.028*
*Eating habits*				
Sweet eater	130 (17.7%)	101 (17.9%)	29 (17.0%)	.872
Night eater	68 (9.24%)	55 (9.73%)	13 (7.60%)	.488
Emotional eater	41 (5.57%)	35 (6.19%)	6 (3.51%)	.250
Binge eater	32 (4.35%)	25 (4.42%)	7 (4.09%)	1.000
*Smoking status*				.593
Nonsmoker	14 (3.44%)	11 (3.77%)	3 (2.61%)	
Current smoker	181 (44.5%)	127 (43.5%)	54 (47.0%)	
Ex-smoker	7 (1.72%)	6 (2.05%)	1 (0.87%)	
Secondhand smoker	46 (11.3%)	37 (12.7%)	9 (7.83%)	

GERD = gastroesophageal reflux disease, PCOS = polycystic ovarian syndrome.

* *P*-value significant if <.05.

One hundred seventy-one readmissions were reported from a total of 736 patients, accounting for 23.23% readmission rate. Of those readmissions, 82.5% underwent LSG procedure.

### 3.2. Predictive factors for readmission

Multivariant logistic regression analysis outcomes are illustrated in Table 3. The results of the analysis showed that RYGB had higher readmission rates (*P*-value = .003, odds ratio [OR] 2.14, 95% confidence interval [CI] 1.32–3.49). Furthermore, both depression (*P*-value = .028, OR 6.49, 95% CI 1.18–52.9) and preoperative BMI (*P*-value = .011, OR 1.03, 95% CI 1.01–1.05) were independent risk factors for readmission.

**Table 3:** Multi-logistic regression model: predictors of readmission.

	Total (n)	*P*-value	Characteristics	Total (n)	*P*-value
Gender (female)	561	.037	Hypertension	140	1.000
Age at time of surgery		.026*	Ischemic heart disease	8	.142
Pre-op BMI	45.1	.011*	Deep vein thrombosis	6	.689
Current BMI	31.1	.795	Obstructive sleep apnea	139	.944
Lowest weight after surgery	77.8	.696	Diabetes	81	1.000
Type of surgery	81 (RYGB)	.003*	Asthma	33	.192
Drain inserted	8	.093	Dyspnea	30	.499
Blood transfusion	3	.554	Thyroid disease	58	.113
Poor oral intake	16	.383	Peptic ulcer disease	4	.232
Sweet eating	130	.872	GERD	81	.639
Binge eating	32	1.000	PCOS	40	.396
Emotional eating	41	.250	Depression	6	.028*
Night eating	68	.488			

BMI = body mass index, RYGB = Laparoscopic Roux-en-Y Gastric Bypass, GERD = gastroesophageal reflux disease, PCOS = polycystic ovarian syndrome.

* *P*-value significant if <.05.

### 3.3. Readmission rates and its causes

The analysis demonstrated a total readmission rate of 23.23% after bariatric surgery (LSG and RYGB) in a single Jordanian center. The 30-day readmission rate was 6.62%.

**Figure 1.**
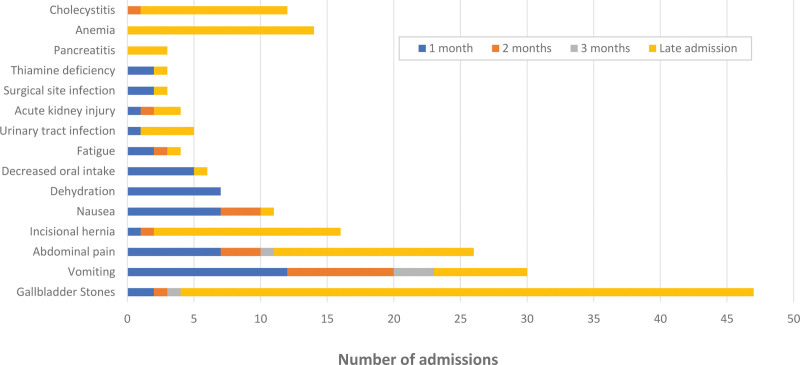
This figure illustrates the various causes of readmission following bariatric surgery, categorized into 5 distinct time frames: within 1 month, 2 months, 3 months and late admission (beyond 3 months). The data highlights the most common reasons for early and late readmissions.

There were several causes identified for readmission of patients after their operation. Each cause identified was further subcategorized into 5 distinct groups based on their timeline postoperatively: 1 month, 2 months, 3 months, late admission (>3 months) and multiple admission. A detailed summary of all causes identified grouped into timelines are shown in Figure 1.

**Figure 2.**
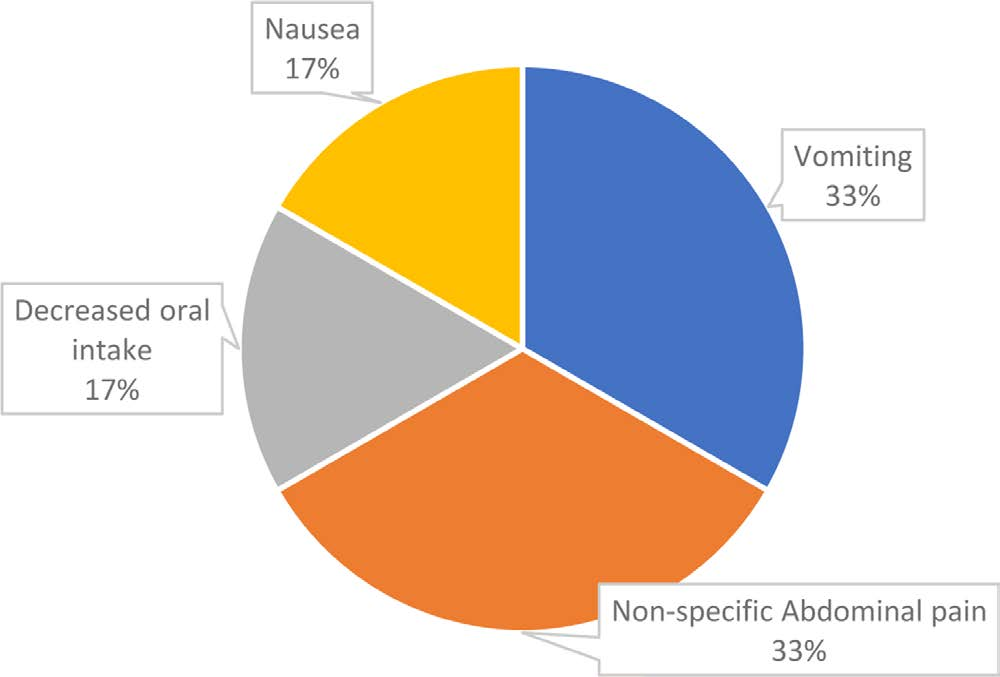
Although only 3.50% of patients required multiple readmissions, this figure illustrates 4 distinct gastrointestinal causes contributing to these readmissions.

Early readmission rate (<90 days postoperatively) was 9.90%. The most common causes of readmission within the first 3 months were vomiting (13.45%), nonspecific abdominal pain (6.42%), nausea (5.84%), and dehydration (4.09%). Among those who required late readmission (>90 days postoperatively), gallbladder stones were the highest cause identified (25.1%), followed by nonspecific abdominal pain (8.77%), incisional hernia (8.19%), symptomatic anemia (8.19%), and cholecystitis (6.43%). All patients who were labeled as having nonspecific abdominal pain were carefully evaluated for gallstone disease and internal hernias. Although a minority of patients required multiple admissions (3.50%), 4 different gastrointestinal causes contributed to this, illustrated in Figure 2.

## 4. Discussion

Bariatric surgery is currently one of the commonest surgeries performed worldwide and therefore subsequent representation and readmission into hospitals remains expected. Readmission rates might vary considerably due to the difference in the predominant type of surgery performed in different centers. Several international studies assessed the rate of hospital readmissions within 90 days and at 1-year follow-up, ranging from 4.1% to 20.5% and 4.75% to 16.6%, respectively.^[13,14]^ Moreover, in a large meta-analysis study that assessed a large cohort, reported a readmission rate range from 0.6% to 20.8% after LSG and 2.4% to 4.0% after RYGB.^[15]^

Several causes are attributed to hospital readmission described in the literature following bariatric surgeries. In the majority of cases postoperatively, it was mainly due to gastrointestinal symptoms of nausea, vomiting (and its sequelae such as dehydration), and abdominal pain (mostly secondary to gallbladder stones and incisional hernia), weather early, late or multiple readmissions. This was similarly described in previously published papers on readmission following bariatric surgery.^[16,17]^ For that reason, there should be considerations when discharging bariatric patients postoperatively. A detailed explanation and instructions, as well as a list of appropriate medication, could be provided to patients postoperatively to aim and reduce the number of readmissions. This can be in the form of a leaflet or information booklet. Similar concepts are found within the guidelines of some bariatric enhanced recovery programs.^[18]^

Specifically for late readmission (>90 days postoperatively), there was a clear association with biliary complications. This raises the question of concomitant cholecystectomy alongside the initial bariatric surgery (either LSG or RYGB). However, a recent systematic review and meta-analysis described a higher postoperative complication rate with concomitant cholecystectomy,^[19]^ and little evidence support improvement in the long-term patient outcomes.

Independent predictive or risk factors were previously described in literature such as diabetes, chronic obstructive pulmonary disease, and hypertension.^[20]^ Our analysis confirm the type of procedure performed, preoperative BMI and depression were all risk factors for readmission. Previous studies have shown that a baseline or previous diagnosis of depression contributes significantly to 30-day readmission rates following bariatric surgery.^[21]^

## 5. Conclusion

The rate of readmission for the 2 most common bariatric surgeries performed in our center within 30-days was 6.62%. The overall readmission rate was 23.23%. The type of bariatric procedure performed preoperative BMI and depression all were independent risk factors for readmission. The most common causes of early readmission within 3 months were nausea, vomiting, and dehydration. Late readmission was mostly due to gallbladder stones and their complications.

## 6. Strengths and limitations

To the best of our knowledge, this study is the first in Jordan to analyze readmission after bariatric surgeries. Moreover, our findings make an important contribution to the growing literature in the field of factors affecting readmission after bariatric surgery. Main limitations are due to the nature of the study being a single center, with a lower proportion of RYGB procedures performed.

## Author contributions

**Conceptualization:** Mohammad Rashdan.

**Data curation:** Shahed Halaseh, Lina Alqirem, Tabarak Alsaadi, Jood Ahmad, Aseel Sabbagh, Faris Haddad, Yasser Algharrawi.

**Methodology:** Bana Al-Mikhi, Shereen Sha'bin, Lina Alqirem, Tabarak Alsaadi, Jood Ahmad, Aseel Sabbagh, Faris Haddad, Yasser Algharrawi.

**Supervision:** Mohammad Rashdan, Mohammad N. Nofal.

**Validation:** Mohammad Salameh, Laith Alghazawi.

**Writing – original draft:** Shahed Halaseh.

**Writing – review & editing:** Lana Al-Sabe.

Critical rivision of the manuscript: Lana Al-Sabe.
